# Learning important features from multi-view data to predict drug side effects

**DOI:** 10.1186/s13321-019-0402-3

**Published:** 2019-12-16

**Authors:** Xujun Liang, Pengfei Zhang, Jun Li, Ying Fu, Lingzhi Qu, Yongheng Chen, Zhuchu Chen

**Affiliations:** NHC Key Laboratory of Cancer Proteomics, Xiangya Hospital, Central South University, XiangYa Road, Changsha, China

**Keywords:** Side effect prediction, Heterogeneous data integration, Feature selection

## Abstract

The problem of drug side effects is one of the most crucial issues in pharmacological development. As there are many limitations in current experimental and clinical methods for detecting side effects, a lot of computational algorithms have been developed to predict side effects with different types of drug information. However, there is still a lack of methods which could integrate heterogeneous data to predict side effects and select important features at the same time. Here, we propose a novel computational framework based on multi-view and multi-label learning for side effect prediction. Four different types of drug features are collected and graph model is constructed from each feature profile. After that, all the single view graphs are combined to regularize the linear regression functions which describe the relationships between drug features and side effect labels. L1 penalties are imposed on the regression coefficient matrices in order to select features relevant to side effects. Additionally, the correlations between side effect labels are also incorporated into the model by graph Laplacian regularization. The experimental results show that the proposed method could not only provide more accurate prediction for side effects but also select drug features related to side effects from heterogeneous data. Some case studies are also supplied to illustrate the utility of our method for prediction of drug side effects.

## Introduction

The safety assessment of candidate chemical compounds is essential for drug development. Detection of serious adverse effects of drugs in preclinical tests or clinical trials is one of the major reasons for the failure of drug development [1]. Furthermore, some side effects are only reported in postmarket surveillance, in which situation serious consequences such as hospitalizations and deaths may be caused by adverse drug reactions [2]. As the performance of traditional methods for side effect detection is limited and the cost of these methods is expensive, there is a great need for developing new approaches that can effectively reveal drug side effects.

Computational approaches have been developed to study various pharmacological problems such as drug repositioning [3–5]. It is also demonstrated that in silico methods could be regarded as complementary or alternative ways to test drug toxicity and predict side effects [6, 7]. Recently, several computational methods utilizing diverse drug related information have been proposed for side effect prediction. Chemical structures of compounds have been conventionally used to predict side effects [8]. For example, Xu et al. employed a deep learning method to encode the chemical structures of drugs and predicted drug-induced liver injury [9]. Atias et al. systematically predicted multiple side effects with chemical structure features by canonical correlation analysis (CCA) [10]. Besides chemical information, biological knowledge of drugs is also useful for predicting side effects. Using drug-protein interactions as input, Mizutani et al. proposed a side effect prediction method based on sparse CCA (SCCA) [11]. Fukuzaki et al. predicted side effects by mapping the targets of drugs to biological pathways [12]. In another work, drug-induced gene expression changes were summarized as biological process terms to predict side effects [13]. Moreover, it’s reasonable to presume that integrating chemical and biological information will help boost the accuracy of side effect prediction. For example, Yamanishi *et al*. predicted side effects by integrating chemical structures and target protein data of drugs [14]. Wang et al. prioritized drug side effects by combining chemical structures and gene expression changes [15]. There are also some studies that include other information such as phenotypic data of drugs to predict side effects [16, 17].

Computational methods are also developed to discover the drug features closely related to side effects. SCCA based methods were proposed to reveal chemical fragments and target proteins related to side effects [11, 18]. Xiao et al. suggested a latent Dirichlet allocation model to learn the relations between drug structures and side effects [19]. Kuhn et al. related the known drug target proteins to side effects by enrichment analysis [20]. Iwata et al. associated protein domains with side effects by sparse classifiers [21]. Chen et al. inferred the associations between proteins and side effects by random walk on a heterogeneous network [22].

Previous computational approaches have demonstrated their ability to predict drug side effects and reveal relevant features. However, there are still some limitations of existing methods. Firstly, although various types of drug features have been utilized for side effect prediction, how to effectively retrieve the complementary information from multiple sources of data is still an open problem. Secondly, some side effects relate to the same group of drugs, these side effects may have a similar molecular basis. How to utilize the correlations between side effect labels to promote the prediction performance is still not fully explored. Thirdly, the dimensions of drug features are usually high. Selecting informative drug features could alleviate the negative impact of the high dimension features on the predictive model and may hint at the molecular basis of side effects. Diverse types of drug characteristics could relate to the occurrence of side effects [21], but few methods are capable of selecting useful features from multiple data sources collectively. The influence of side effect label correlations on feature selection has also not been well considered. This will impair the performance of data fusion model.

Multiple types of drug features could be integrated by multi-view learning. Multi-view learning aims at incorporating heterogeneous data in a unified model to retrieve complementary information and improve predictive performance [23]. Multi-view learning has also been applied in previous pharmacological studies. Zhang et al. have predicted drug target interactions by integrating multi-view network data [24]. In our previous work, a multi-view learning method was proposed for prediction of drug-disease associations [3]. In the study of Yamanishi et al. they used multiple kernel learning to predict drug side effects [14]. On the other hand, the correlations between side effect labels could be exploited by multi-label learning. Multi-label learning deals with the classification problems in which samples are associated with multiple labels. How to use the correlations between labels to improve classification accuracy is a major task of multi-label learning [25]. Multi-label learning has been applied to various problems, such as protein function and subcellular localization prediction [26, 27]. Besides, for multi-label classification, each class label may be discriminated by some special characteristics of its own. These discriminative characteristics are denoted as label specific features. Selection of label specific features could be of benefit to multi-label classification [28]. Multi-view learning and multi-label learning could handle different aspects of side effect prediction problem, thus combining these two methods could explore feature heterogeneity and label correlations simultaneously.

Graph Laplacian regularization is one of the manifold learning algorithms and has many applications in machine learning [29]. It looks for a sufficiently smooth distribution of data in a low-dimensional manifold and encourages locality preserving properties of the learning model [30]. For multi-view data, the graph Laplacian based algorithms employ a neighbourhood graph to capture the local geometry of each view, then all the graphs are aligned to extract the complementary information [31]. Shi et al. utilized graph Laplacian regularization to integrate multi-view data and proposed a semi-supervised sparse feature selection method [32]. Our previous work on drug-disease association prediction also utilized the multi-view Laplacian regularization [3]. For multi-label classification, the correlations among labels can also be encoded as a graph. For example, in the work of Mojoo et al. they introduced graph Laplacian regularization to represent the co-occurrence dependency between image tags [33].

After realizing the limitations of existing methods, we intend to investigate the problem of side effect prediction by fusing the ideas of multi-view and multi-label learning. For this purpose, graph Laplacian regularization is employed to model both the relationships between heterogeneous features and the correlations between labels. Similar to the previous works [3, 32], the complementary information from multiple drug feature profiles is explored by combining all view-dependent graph models. The correlations between side effect labels are introduced as an additional graph Laplacian regularization term. Furthermore, linear regression with L1-norm penalty is incorporated into the model to get label specific features from different feature profiles, which is similar to the graph constrained Lasso [34]. An iterative algorithm is proposed to solve the optimization problem of the model. Then, four different feature profiles, including chemical substructures of drugs, protein domains and gene ontology terms of drug targets and drug-induced gene expression changes are collected. These heterogeneous features are integrated by our method to predict side effects of drugs. The performance of our method is compared with several existing methods. We also illustrate the predictive capability of the proposed method with some case studies and examine the selected features. The results show that our method outperforms the compared methods.

## Methods

### Data collection

The side effects of drugs were retrieved from SIDER [35]. The chemical structures of drugs were derived from PubChem [36]. The protein targets of drugs were obtained from DrugBank [37], only target proteins related to human were kept. The protein domains of the targets were collected from InterPro [38] and gene ontology information (only using biological process terms) was extracted from Uniprot [39]. The gene expression data of drugs in the LINCS L1000 project were downloaded [40]. Finally, 501 drugs with all above information were kept for the following analysis and model construction. The DrugBank identities of these drugs and the count of side effect labels for each drug could be found at the (Additional file 2: Table S1). The identifiers and names of the features are supplied at (Additional file 2: Table S2). We also obtained the off-label side effects recorded in FDA Adverse Event Report System (FAERS) from the previous work [15]. There are 106 drugs with all types of features and off-label side effects in FAERS but without any records in SIDER. 294 drugs are present in both SIDER data and FAERS data, and these drugs have additional 65873 drug-side effect associations in FAERS data. The information of these drugs is available at (Additional file 2: Tables S3 and S4).

### Data preprocessing and drug feature matrices building

For chemical substructures of drugs, the fingerprints defined by PubChem were calculated using PyBioMed [41]. The chemical substructure matrix is built using the binary fingerprints (881 bits in total). With the target information of drugs, two binary feature matrices are constructed. The target protein domain matrix is a binary matrix in which the elements are 1 if the targets of drugs have the corresponding protein domains or 0 otherwise. There are 1307 unique protein domain features. In the target gene ontology matrix, the elements are set to 1 if the targets of drugs are annotated with the gene ontology terms. There are 3336 unique gene ontology features. For drug induced gene expression changes from LINCS L1000 project, only the values of the 978 landmark genes were kept. If the absolute value of the moderated z-score for a gene signature is bigger than 2, the signature value is set to 1 or − 1 depending on the original sign of the moderated z-score, otherwise the signature value is set to 0. After that, the elements of the gene expression matrix are evaluated as the averages of signature values of each gene for each drug. Through above data processing, four feature matrices $X_p\in \mathbb {R}^{n\times d_p}$ of drugs are obtained, where *n* is the number of drugs, $d_p$ is the number of features in the *p*th feature matrix.

The relationships between drugs and side effects were extracted from the ‘meddra_all_se’ file of SIDER and only preferred terms for side effects were kept. There are 3260 side effect labels in total. If there is a record of the relationship between drug i and side effect j, we set the element in the ith row and jth column of the label matrix $Y\in \mathbb {R}^{n\times l}$ to 1, otherwise set the element to 0, where *l* is the number of side effects.

### Problem formalization

In this work, we plan to construct a computational model which could predict side effects of drugs and select label specific features by integrating multiple types of drug data $\{X_p\}$. Firstly, the formulation of our method, which is named as multi-view Laplacian regularized sparse learning (Multi-LRSL), is introduced. Then an optimization algorithm for solving Multi-LRSL is presented.

#### Multi-LRSL model

*Predicting side effects with special drug features* We assume that different types of drug features are complementary to each other and could be exploited to predict side effects. Moreover, each side effect should be only associated with a subset of features from different feature profiles. That is, the drug features relevant to side effects are sparse. As a result, we model the relationships between drug features and side effects by least square loss, and use $L_1$-norm to regularize the coefficient matrices:

1 $$\begin{aligned} \min _{G_p, F}\frac{\mu }{2}\sum _{p=1}^{m}{\Vert X_pG_p-F\Vert _F^2} + \beta \sum _{p=1}^{m}{\Vert G_p\Vert _1} \end{aligned}$$

where $\Vert \cdot \Vert _F$ is the Frobenius norm, $\mu$ and $\beta$ are the model parameters. $G_p$ represents the regression coefficient matrix for the *p*th feature profile, *m* is the number of feature types, *F* is the predicted side effect label matrix and contains continuous values. In the label matrix *Y*, the elements are set to 1 for positive labels and 0 for negative or unobserved labels. *F* should be similar but not identical to *Y* because *Y* may contain some missing and noisy values. The elements of F could be ranked, and the bigger values imply possible positive labels and the smaller values imply possible negative labels. In the second term, the $L_1$-norm with the parameter $\beta$ controls the sparsity of side effect related features. The non-zero elements in the *j*th column of $G_p$ are the relevant features for the *j*th side effect.

*Preserving the local structure of different feature space in the side effect label space* We assume that drugs with similar features should have similar side effect labels. This is known as the smoothness assumption [42]. For each type of drug features, a pairwise drug similarity matrix is calculated, then the k-nearest neighbour (knn) graph $S_p$ is constructed:

2 $$\begin{aligned} S_p(i,j)=\left\{ \begin{array}{lll} sim(X_p(i,:),X_p(j,:)) &\quad \text {if } X_p(j,:) \text { is the k-nearest } \text {neighbor of } X_p(i,:)\\ 0 &\quad \text {otherwise,} \end{array}\right. \end{aligned}$$

where $X_p(i,:)$ and $X_p(j,:)$ are the row vectors of the *p*th feature matrix. In this work, we use cosine similarity for all feature profiles and set $k = \lfloor 0.01n\rfloor$. The rows of *F* are the predicted side effect labels for drugs. As the result of the smoothness assumption, we get the following formula:

3 $$\begin{aligned} \min _{F}\sum _{i,j}^{n}\Vert F(i,:)-F(j,:)\Vert _2^{2}S_{p}(i,j) \end{aligned}$$

This means that drugs with similar features in the *p*th feature profile should have similar predicted labels. The local geometry of the feature space is preserved in the predicted label space. The above formula could be rewritten as:

4 $$\begin{aligned} \min _{F}Tr(F^{T}L_{p}F) \end{aligned}$$

where the Laplacian matrix $L_p$ is defined as $L_p = D_p - S_p$, and $D_p(i,i) = \sum _j^nS_p(i,j)$. To explore the complementary information in different types of drug features, multi-view Laplacian regularization is adopted as in the previous studies [3, 32]. The graph Laplacian matrices of different feature profiles are combined using a weight vector $\theta \in \mathbb {R}^{m\times 1}$. In addition, the predicted label matrix *F* should not only be smooth on the feature space but also be consistent with the original label matrix *Y*. These considerations give the following formula:

5 $$\begin{aligned}&\min _{F,\theta}\frac{1}{2}Tr\big (\sum _{p=1}^m\theta _p^\gamma F^TL_pF\big ) + \frac{1}{2}\Vert F - Y\Vert _F^2\nonumber \\ & \quad s.t.\ \theta > 0,\ \sum _{p=1}^m{\theta _p}=1 \end{aligned}$$

In the above formula, the weights of graph Laplacian matrices mean that different types of features have different contributions to side effect prediction. The weight vector $\theta$ could also be learned by optimization. The parameter $\gamma >1$ is introduced to keep the elements of $\theta$ from equalling zero. This will prevent the most predictive feature profile taking all the weights [43]. In this way, the correlated and complementary information from multiple data sources could be combined and transferred to predicted label space.

*Incorporating side effect label correlations* Next, under the assumption that strongly correlated side effect labels will share more drug features, it is desirable to incorporate label correlations into our model. According to Eq. (1), the columns of the coefficient matrix $G_p$ represent the drug features associated with side effects. For highly correlated side effect labels, the corresponding column vectors in $G_p$ should have great similarity. Similar to the consideration for the relationship between drug similarity and side effect similarity, we use Laplacian graph to represent the relationships between label correlations and feature sharing. The cosine similarity is employed to describe the correlations between side effect labels. A knn graph $R_0$ is constructed based on label correlations. As mentioned above, the known side effect labels are usually incomplete and noisy, we intend to refine the correlation graph while learning the feature coefficients. Then the graph regularization for label correlations is formulated as:

6 $$\begin{aligned}&\min _{G_p,R}\frac{\lambda }{2}\sum _{p=1}^{m}{Tr(G_p(D_R-R)}G_p^T) + \frac{\alpha }{2}\Vert R - R_0\Vert _F^2\nonumber \\ & \quad s.t.\ R_{ij}=R_{ji}\ge 0 \end{aligned}$$

where *R* is the refined correlation graph, $D_R$ is degree matrix of *R*, $D_R-R$ is the Laplacian matrix. ${Tr(G_p(D_R-R)G_p^T)}$ is equal to $\sum _{i,j}^l\Vert G_p(:,i)-G_p(:,j)\Vert _2^2R(i,j)$. As a result, this term encourages a pair of highly correlated side effect labels to be associated with similar drug feature coefficients. $\alpha$ is a positive parameter which controls the extent of consistency between the refined correlation graph and the original correlation graph. The parameter $\lambda$ controls the impact of label correlations on the similarities of feature coefficients.

*The final objective function* After integrating the above formulae, the final objective function takes the following form:

7 $$\begin{aligned}&\mathop {min}\limits _{F, G_p, R, \theta} \frac{1}{2}\Vert F-Y\Vert _F^2 + \frac{1}{2}Tr(F^TLF) \nonumber \\&\qquad + \frac{\mu }{2} \sum _{p=1}^{m}{\Vert X_pG_p-F\Vert _F^2 } \nonumber \\&\qquad + \frac{\lambda }{2}\sum _{p=1}^{m}{Tr(G_p(D_R -R)G_p^T)} + \frac{\alpha }{2}\Vert R-R_0\Vert _F^2 \nonumber \\&\qquad +\beta \sum _{p=1}^{m}{\Vert G_p\Vert _1} \nonumber \\&\quad s.t.\ L = \sum _{p=1}^m{\theta _p^\gamma L_p}, \ \sum _{p=1}^m{\theta _p} = 1,\ 0< \theta _p < 1, R_{ij}=R_{ji}\ge 0 \end{aligned}$$

The first three terms in above formula give a flexible manifold learning framework [44]. The fourth and fifth terms account for label correlations. Together with the last $L_1$ penalties, these terms form a graph constrained Lasso [34].

#### Optimization

Here, an alternating approach is proposed for optimizing the objective function (7).

*Update* *F* First, $G_p$, *R* and $\theta$ are fixed, the derivative of objective function with respect to *F* is set to 0. The closed form solution of *F* is:

8 $$\begin{aligned} F=PQ \end{aligned}$$

where

9 $$\begin{aligned} P = \bigl ((1+m\mu )I + L\bigr )^{-1} \end{aligned}$$

and

10 $$\begin{aligned} Q = Y + \mu \sum _{p=1}^m{X_pG_p} \end{aligned}$$

*Update* *R* Then *F*, $\theta$ and $G_p$ are fixed, *R* is optimized by multiplicative updates [45]. The Lagrangian function of R is:

11 $$\begin{aligned} \mathcal {L}(R)=\frac{\lambda }{2}\sum _{p=1}^{m}{Tr(G_p(D_R -R)G_p^T)} + \frac{\alpha }{2}\Vert R-R_0\Vert _F^2 - Tr(\Gamma R) \end{aligned}$$

$\Gamma$ is Lagrangian multiplier. Differentiating the above formula with respect to *R*:

12 $$\begin{aligned} \frac{\partial \mathcal {L}}{R} = \frac{\lambda }{2}\sum _{p=1}^{m}{A_p}-\frac{\lambda }{2}\sum _{p=1}^{m}{B_p} + \alpha (R - R_0) - \Gamma \end{aligned}$$

where

13 $$\begin{aligned} A_p & = {} diag(G_p^T G_p)J+Jdiag(G_p^TG_p) - diag(G_p^TG_p) \end{aligned}$$

14 $$\begin{aligned} B_p & = {} 2G_p^TG_p - diag(G_p^TG_p) \end{aligned}$$

$J_p$ is a $l\times l$ matrix of all 1’s. Using the KKT condition, $\Gamma (i,j)R(i,j)=0$, then:

15 $$\begin{aligned} R(i,j) \leftarrow R(i,j) \sqrt{\frac{\frac{\lambda }{2}\sum _{p=1}^{m}{(B_p)^+} + \alpha R_0}{\alpha R + \frac{\lambda }{2}\sum _{p=1}^{m}{(B_p)^-} + \frac{\lambda }{2}\sum _{p=1}^{m}{A_p}}}(i,j) \end{aligned}$$

where $$(B_p)^+=(|B_p|+B_p)/2$$ and $$(B_p)^-=(|B_p|-B_p)/2$$

*Update* $G_p$ Next, fixing the other variables except $G_p$, substituting *F* with *P*, *Q* and ignoring unrelated terms in the objective function (7), we get:

16 $$\begin{aligned}&\mathop {min}\limits _{G_p} F(G_p) =\frac{1}{2}Tr\bigl (\sum _{p=1}^m{G_p^TX_p^T(\mu I-\mu ^2P^T)X_pG_p} \bigr )\nonumber \\&\quad - \mu Tr\bigl ( Y^TP^T\sum _{p=1}^m{X_pG_p}\bigr )\nonumber \\&\quad - \frac{\mu ^2}{2}Tr\bigl (\sum _{p=1}^m{\sum _{q\ne p}^m{G_p^TX_p^TP^TX_qG_q}}\bigr ) \nonumber \\&\quad + \frac{\lambda }{2}\sum _{p=1}^{m}{Tr(G_p(D_R -R)G_p^T)} + \beta \sum _{p=1}^{m}{\Vert G_p\Vert _1} \end{aligned}$$

Due to the $L_1$-norm regularization terms, the above Eq. (16) is convex but not smooth, so the accelerated proximal gradient method is utilized to solve it. For a special *p*, Let

17 $$\begin{aligned} f(G_{p}) =&\frac{1}{2}Tr\bigl (G_{p}^TX_{p}^T(\mu I-\mu ^2P^T)X_{p}G_{p} \bigr )- \mu Tr\bigl ( Y^TP^TX_{p}G_{p}\bigr )\nonumber \\&- \mu ^2Tr\bigl (\sum _{q\ne p}^m{G_{p}^TX_{p}^TP^TX_{q}G_{q}}\bigr ) + \frac{\lambda }{2}Tr\left(G_{p}(D_R-R)G_{p}^T\right) \end{aligned}$$

and

18 $$\begin{aligned} g(G_{p}) = \beta \Vert G_{p}\Vert _1 \end{aligned}$$

Next, the proximal gradient algorithm is employed to minimize a sequence of quadratic approximations of $F(G_{p})$:

19 $$\begin{aligned} \mathop {min}\limits _{G_{p}}f(\tilde{G}_{p}^{(k)}) + \langle \nabla f(\tilde{G}_{p}^{(k)}), G_{p}-\tilde{G}_{p}^{(k)}\rangle + \frac{L_f}{2}\Vert G_{p} -\tilde{G}_{p}^{(k)}\Vert _F^2 + g(G_{p}) \end{aligned}$$

In the above formula, $L_f$ is Lipschitz constant. $\tilde{G}_{p}^{(k)}=G_{p}^{(k)} + \frac{t_{k-1} - 1}{t_{k}}\bigl (G_{p}^{(k)} - G_{p}^{(k-1)}\bigr )$, and $t^2_{k}-t_{k}\le t_{k-1}^2$. According to [46] it could improve the convergence rate. Let $Z_{p}^{(k)} = \tilde{G}_{p}^{(k)} - \frac{1}{L_f}\nabla f(\tilde{G}_{p}^{(k)})$, (19) could be rewritten as:

20 $$\begin{aligned} \mathop {min}\limits _{G_{p}}\frac{L_f}{2}\Vert G_{p} - Z_{p}^{(k)}\Vert _F^2 + g(G_{p}) \end{aligned}$$

then, $G_{p}$ could be optimized by

21 $$\begin{aligned} G_{p} & = {} \mathop {argmin}\limits _{G_{p}}\frac{1}{2}\Vert G_{p} - Z_{p}^{(k)}\Vert _F^2 + \frac{\beta }{L_f}\Vert G_{p}\Vert _1 \nonumber \\ & = {} S_{\frac{\beta }{L_f}}(Z^{(k)}_{p}) \end{aligned}$$

where $S_{\frac{\beta }{L_f}}$ is the soft-thresholding operator.

*Update* $\theta _p$ Finally, *F*, *R* and $G_p$ are fixed and $\theta _p$ is renewed

22 $$\begin{aligned} \theta _p = \frac{\Bigl (\frac{1}{Tr(F^TL_pF)}\Bigr )^{\frac{1}{\gamma -1}}}{\sum _{p=1}^m{\Bigl (\frac{1}{Tr(F^TL_pF)}\Bigr )^\frac{1}{\gamma -1}}} \end{aligned}$$

Through the above updating steps, the objective function (7) is solved. The outline of multi-LRSL is shown in Algorithm 1. After training the model, we obtain the coefficient matrices $G_p$s and the predicted label matrix *F*. For new drugs, the features $X_p^{new}$are collected, and the side effects are predicted by $\sum _{p=1}^{m}\theta _{p}X_{p}^{new}G_{p}$. The elements of *F* could be considered as the confidence of the predicted associations between drugs and side effects. The missing side effect labels in the training data could be inferred from *F*. For that, the values corresponding to the known drug-side effect associations in *F* are excluded, then the rest values are ranked in descending order. The top-ranked values suggest the most possible drug-side effect associations that are missing in the original data.

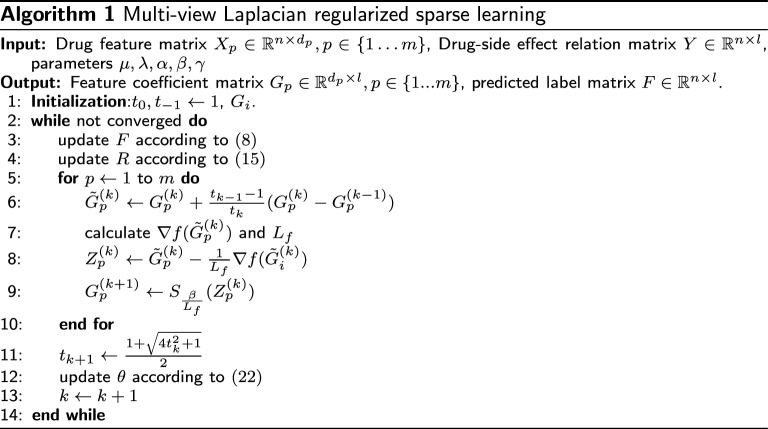



### Performance evaluation and comparison methods

To evaluate the performance of our algorithm for side effect prediction, fivefold cross-validation was carried out in this work. We used six performance metrics implemented by scikit-learn [47]. First, the area under the receiver operating characteristic curve (AUC) score was employed. The receiver operating characteristic (ROC) curve is plotted with true positive rate (TPR) against the false positive rate (FPR).

23 $$\begin{aligned} TPR= & {} \frac{TP}{TP+FN} \end{aligned}$$

24 $$\begin{aligned} FPR= & {} \frac{FP}{FP+TN} \end{aligned}$$

where TP is true positive, FN is false negative, FP is false positive, TN is true negative. The AUC score is the area under the ROC curve. Depending on which type of averaging was performed, three kinds of AUC scores were calculated. Sample-AUC considers the average AUC score for each drug, macro-AUC calculates AUC scores for each side effect label and finds the mean, micro-AUC takes all known drug-side effect pairs as a positive label.

Second, three metrics special for multi-label classification were estimated. Coverage error represents the average number of side effect labels to be included in order to predict all true labels. Given the truth labels of test data $Y_{test}\in \mathbb {R}^{n_{test}\times l}$, the score matrix from prediction method is $\hat{Y}$, the coverage error is:

25 $$\begin{aligned} Coverage\ error = \frac{1}{n_{test}}\sum _{i=1}^{n_{test}}\max _{j:Y_{test}(i,j)=1}{rank(i,j)}-1 \end{aligned}$$

where $rank(i,j) = |\{k:\hat{Y}(i,k)\ge \hat{Y}(i,j)\}|$, $|\cdot |$ is the number of elements in the set. $n_{test}$ is the number of test drugs.

Ranking loss is the average number of label pairs that are incorrectly ordered:

26 $$\begin{aligned}&ranking\ loss\nonumber \\&\quad =\frac{1}{n_{test}}\sum _{i=1}^{n_{test}}\frac{1}{\Vert Y(i,:)\Vert _0(l-\Vert Y(i,:)\Vert _0)}\Big |\Big \{(k,j):\hat{Y}(i,k)\le \hat{Y}(i,j),\nonumber \\&\qquad Y(i,k)=1,Y(i,j)=0\Big \}\Big | \end{aligned}$$

where $\Vert \cdot \Vert _0$ is the number of non-zero elements.

Label ranking average precision (LRAP) finds the average fraction of the true labels in the highly ranked labels produced by a predictive method:

27 $$\begin{aligned} LRAP & = {} \frac{1}{n_{test}}\sum _{i=1}^{n_{test}}\frac{1}{\Vert Y(i,:)\Vert _0}\nonumber \\&\sum _{j:Y(i,j)=1}\frac{|\{k:Y(i,k)=1,\hat{Y}(i,k)\ge \hat{Y}(i,j)\}|}{rank(i,j)} \end{aligned}$$

For our method, the score matrix $\hat{Y}=\sum _{p=1}^{m}\theta _{p}X_{p}^{new}G_{p}$. The truth label matrix $Y_{test}$ was used to calculate the performance metrics in each fold of cross-validation. The experiment was repeated 10 times with different division of data and the averages of the metrics were calculated. We compared our algorithm with the other five computational methods. L1-regularized logistic regression (L1LOG) and L1-regularized support vector machine (L1SVM) are widely used for feature selection and classification. These methods were also applied to inferring the relationships between side effects and target domains [21]. L1LOG and L1SVM were implemented with Liblinear [48]. Principal component regression (PCR) is based on principal component analysis (PCA). It firstly computes the principal components of the feature matrix, then uses some of the components as predictors to build model for prediction. In this work, PCR was implemented to represent another way for dimensionality reduction. SCCA was also demonstrated to be effective for discovering drug features related to side effects [11, 18]. As in the previous work, SCCA was implemented with PMA package [49]. Kernel Regression was used to integrate chemical structures and target proteins to predict side effects in [14]. Here we implemented it with scikit-learn. The parameters of all methods were determined by cross-validation. More details about the implementation of the comparison methods could be found in additional material. The algorithm complexity analysis and the parameter sensitivity analysis of the proposed method could also be found in the additional material.

## Results

### Drugs with similar features have similar side effect labels

In these work, there are 501 drugs, 3260 side effects and 62620 associations between them. The distribution of the associations is shown in Additional file 1: Figure S1. In this section, we validated the basic assumption that the drug features collected from different sources were associated with the side effect labels of drugs. First, we examined the features of the drugs with at least one common side effect. It is noticed that the average similarity of the drugs with common side effects is significantly stronger than the same number of randomly selected drugs without any common side effects across all feature profiles (rank sum test, $\hbox {p-value}<0.001,$ Additional file 1: Figure S2). This suggests that drugs which cause the same side effects share more common features. Next, we calculated the side effect similarities between drugs and divided the drugs into two groups at the median value of similarities. It is showed that drugs with more common side effects also display significantly stronger similarity in all types of features (rank sum test, $\hbox {p-value}<0.001$, Additional file 1: Figure S3). These results imply that similar drugs possess similar side effect labels. To further verify this assumption, we calculated the inner products between the columns of the feature matrix $X_p$ and the columns of the side effect matrix *Y* and utilized these products to represent the relationships between drug features and side effect labels. Then we computed the cosine similarity between side effects using different types of dug features related to them. After that, we built ordinary least squares models which took different feature similarities between side effects as explanatory variables and side effect label correlations as response variables. It is observed that the slopes of these linear models are positive, which means that the feature similarities positively correlate with the correlations of side effect labels. It is also noted that when the values of feature similarity get bigger, the slopes become steeper (Additional file 1: Figure S4). This suggests that the correlations between the drug features and the side effect labels are more obvious in local feature space. All above results demonstrate that the feature profiles employed in this work are associated with the side effects labels, so it is possible to predict the side effects of drugs with these features.

**Fig. 1:**
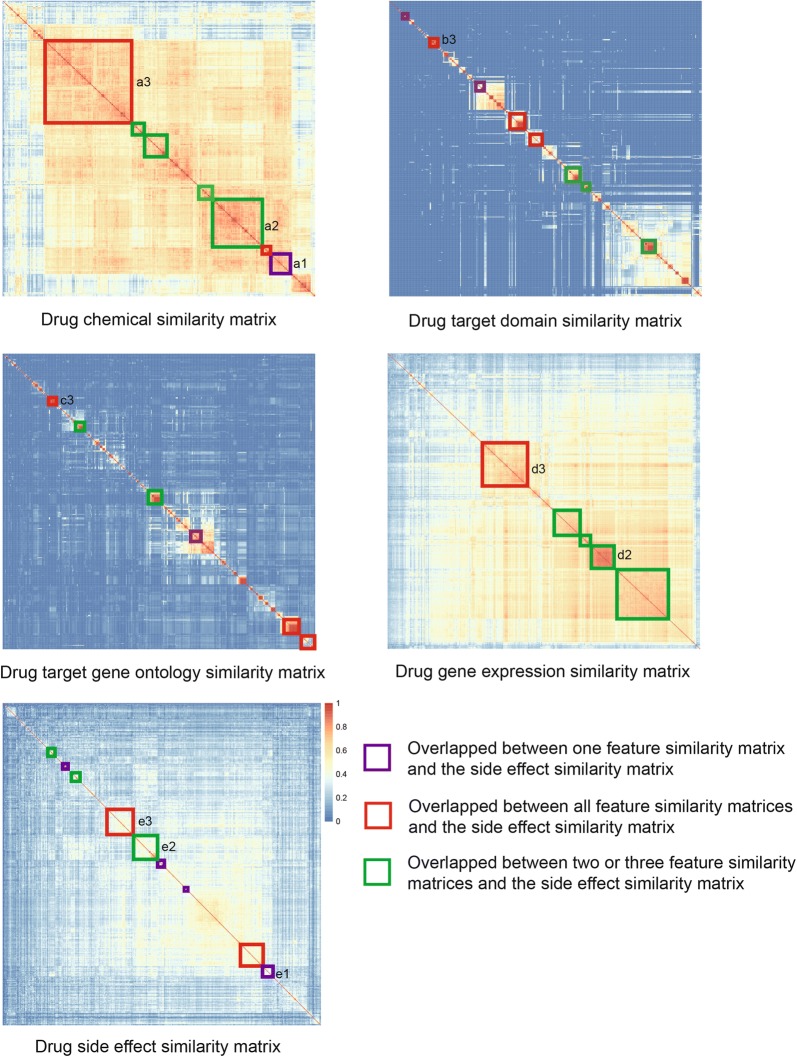
There is both consistent and complementary information related to side effects in different drug feature profiles. Drugs cluster together according to the similarities calculated with different features or side effect labels. The blocks of drugs along the diagonals are identified by the R package ‘dynamicTreeCut’ [67]. The overlaps between the blocks in each feature similarity matrix of drugs and the blocks in side effect similarity matrix of drugs are determined by Fisher’s exact test ($\hbox {p-value}<0.05$). The significantly overlapping blocks are marked by coloured rectangles in the heat-maps. The purple rectangles indicate that the blocks in the side effect similarity matrix of drugs overlap with blocks in one of the feature similarity matrices (for example, block *e1* overlaps with block *a1* in the chemical similarity matrix). The green rectangles indicate that the blocks in the side effect similarity matrix of drugs overlap with blocks in two or three feature similarity matrices (for example, block *e2* overlaps with *a2* in the chemical similarity matrix and block *d2* in the gene expression similarity matrix). The red rectangles indicate that the blocks in the side effect similarity matrix of drugs overlap with blocks in all feature matrices (for example, block *e3* overlaps with block *a3, b3, c3, d3*). The legend indicates the value of similarity, from 0 (blue) to 1 (red)

There are four different types of drug features in this work. It is expected that these feature profiles will provide consistent as well as complementary information for side effect prediction. To explore the consistency and complementarity of these feature profiles, we performed hierarchical cluster analysis according to the drug similarity matrices calculated by the feature profiles and the side effect labels. As show in Fig. 1, there are many blocks along the diagonals of the similarity matrices. These blocks are drugs with strong similarity It is observed that the drugs in some blocks of the feature similarity matrices significantly overlap with the drugs in the blocks of the side effect similarity matrix (Fisher exact test, $\hbox {p-value}<0.05$). The overlapped blocks are marked by coloured rectangles in Fig. 1. Furthermore, for some blocks in the side effect similarity matrix, there are overlapped blocks across more than one feature similarity matrices. It is also found that some blocks in the side effect similarity matrix only overlap with the blocks from just one of the feature similarity matrices. These results indicate that there is both consistent and complementary information in different drug feature profiles, and combination of these feature profiles could be beneficial for side effect prediction. There are also some blocks in the feature similarity matrices which don’t overlap with any blocks in the side effect similarity matrix. It is imply that there are irrelevant drug features to be excluded or missing associations between drugs and side effects.

### Performance comparison for prediction of side effects

To evaluate the performance of the proposed method, we tested if the algorithm could correctly recover the known side effects of drugs. Five-fold cross-validation was performed. Drugs with known side effects from SIDER were divided into five subsets of roughly equal size. Each time one subset of drugs was used as test set while the other four sets were combined as training set. The experiment was repeated 10 times with different division of drugs. In this work, the performance for side effect prediction of the proposed method was compared with other algorithms using six different metrics. L1SVM and L1LOG are two widely used sparse models that could be applied to both classification and feature selection. PCR is a regression technique which could reduce dimensionality and mitigate overfitting. For the task of side effect prediction, the four drug feature matrices were concatenated into a single matrix then this long matrix was used as the input to L1SVM , L1LOG and PCR. For SCCA, the side effect label matrix, together with each feature matrix were used as input. The drug target matrix was also used as the input to SCCA following the previous work [11]. For kernel regression, the kernel similarity matrix for each feature profile was calculated, then the kernel functions were summed to integrate different feature profiles. For the performance metrics used here, larger values of sample-AUC, macro-AUC, micro-AUC and LRAP mean better performance, while smaller values of coverage error and ranking loss denote better performance. Table 1 shows the results of the cross-validation experiment for the comparison algorithms. Overall, the proposed integrative method outperforms all the comparison methods significantly. Furthermore, when just taking one single type of features as input, our method still has better performance in most of the metrics compared to SCCA. It is also observed that the target domain and the target gene ontology features generally show better performance compared to the chemical substructure features and the gene expression features if only one feature matrix is used as the input to our method. As the other information integration method, the kernel regression model also shows some advantages over SCCA. The performance of L1LOG and L1SVM is inferior to the other methods except PCR. This may be partially due to the lack of consideration for the correlations between side effect labels. The performance of PCR is comparable to L1LOG and L1SVM. The results imply that L1-regularization has similar effect with principal components analysis on the performance of side effect prediction.

**Table 1:** Performance comparison of different algorithms for side effect prediction

	Sample-AUC	Macro-AUC	Micro-AUC	LRAP	Coverage error	Ranking loss
L1SVM	0.8524 ± 0.0010	0.6196 ± 0.0056	0.8328 ± 0.0010	0.1941 ± 0.0013	2671 ± 11	0.1483 ± 0.0010
L1LOG	0.8612 ± 0.0010	0.6191 ± 0.0059	0.8418 ± 0.0010	0.2018 ± 0.0015	2562 ± 15	0.1394 ± 0.0010
PCR	0.8824 ± 0.0004	0.5034 ± 0.0033	0.8670 ± 0.0006	0.1890 ± 0.0010	2666 ± 16	0.1233 ± 0.0004
SCCA-chem	0.8500 ± 0.0019	0.5731 ± 0.0045	0.8181 ± 0.0019	0.4008 ± 0.0032	2960 ± 23	0.1507 ± 0.0020
SCCA-domain	0.9144 ± 0.0007	0.6260 ± 0.0055	0.8922 ± 0.0007	0.4757 ± 0.0011	2547 ± 13	0.0863 ± 0.0008
SCCA-GO	0.8911 ± 0.0015	0.6160 ± 0.0057	0.8579 ± 0.0015	0.4509 ± 0.0017	2789 ± 25	0.1097 ± 0.0015
SCCA-expression	0.9076 ± 0.0007	0.5159 ± 0.0031	0.8878 ± 0.0007	0.4488 ± 0.0005	2607 ± 9	0.0941 ± 0.0007
SCCA-target	0.9174 ± 0.0005	0.6159 ± 0.0051	0.8968 ± 0.0005	0.4692 ± 0.0007	2490 ± 11	0.0834 ± 0.0005
Kernel regression	0.9185 ± 0.0005	0.6134 ± 0.0053	0.8992 ± 0.0005	0.4766 ± 0.0007	2448 ± 8	0.0821 ± 0.0005
LRSL-chem	0.9179 ± 0.0003	0.5595 ± 0.0034	0.8976 ± 0.0004	0.4614 ± 0.0005	2583 ± 10	0.0867 ± 0.0005
LRSL-domain	0.9285 ± 0.0005	0.6470 ± 0.0050	0.9104 ± 0.0007	0.4821 ± 0.0005	2174 ± 14	0.0719 ± 0.0005
LRSL-GO	*0.9290 ± 0.0007*	0.6441 ± 0.0043	0.9068 ± 0.0010	*0.4924 ± 0.000*	2255 ± 15	0.0714 ± 0.0007
LRSL-expression	0.9203 ± 0.0004	0.5131 ± 0.0013	0.9008 ± 0.0005	0.4565 ± 0.0006	2198 ± 11	0.0801 ± 0.0004
Multi-LRSL	*0.9295 ± 0.000*	*0.6568 ± 0.0057*	*0.9118 ± 0.0009*	0.4845 ± 0.0006	*2160 ± 13*	*0.0709 ±0.0006*

The metrics are denoted as $\hbox {mean}\pm \hbox {standard deviation}$ . The method taking different types of features as input is indicated in the form of ’method name-feature type’

The best metric values are in italics, and the difference between the best value and the second best value of each metric is significant (student t-test, $\hbox {p-value}<0.05$)

To further illustrate the predictive ability of the propose method, we exploited the drugs and their side effect labels from SIDER to train our model, then used the records of the off-label side effects from FAERS as independent test data. The proposed method still has better predictive performance for the drugs which are only present in FAERS compared to SCCA and kernel regression (Additional file 2: Table S5). Besides these new drugs, FAERS records some novel associations between drugs and side effects which are not present in SIDER. It is also found that the proposed method could better predict these novel drug-side effect associations ($\hbox {micro-AUC}=0.6815$) compared to SCCA and kernel regression model ($\hbox {micro-AUC}= 0.6736$ and 0.6768 respectively). Moreover, because we only use the drugs with all four types of features in the above cross-validation experiment, there are still extra drugs which have side effect labels in SIDER but are not used for model training due to lack of target or gene expression information. The identities of these drugs are available at (Additional file 2: Table S6). We predicted side effects of these extra drugs with the chemical substructure features and calculated the performance metrics. It is noticed that the prediction performance of our model on these drugs is comparable with the cross-validation result (Additional file 2: Table S6). This indicates that the overfitting risk of the proposed model is under control and the model could be generalized well to unseen data.

### Selection of side effect related features

**Fig. 2:**
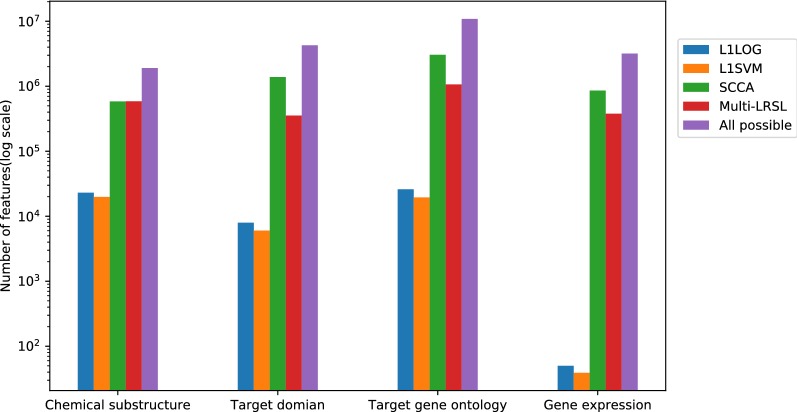
The number of features selected by different methods from different feature profiles

**Fig. 3:**
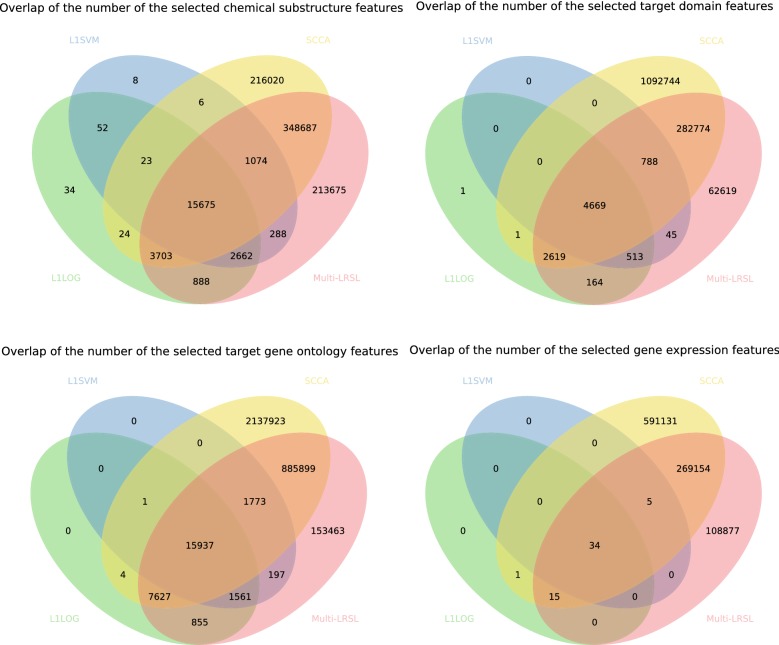
The Venn diagrams show the overlaps of features selected by different methods from different feature profiles

In order to get a predictive model for the side effect prediction problem with high-dimension features, the L1 penalties are added to the model. As a result, our method could not only predict the side effects of drugs but also select the relevant drug features for each side effect. In this section, the feature selection capability of the proposed method was examined and compared with the other three L1-regularization methods: L1LOG, L1SVM and SCCA. The drug features from all feature profiles were selected by different methods. The positively weighted features were kept and thought to be closely related to the corresponding side effect labels. The number of the selected features from each feature profile by each method is shown in Fig. 2. The median numbers of the selected features for each side effect by the proposed method are 161 (chemical substructures), 43 (protein domain), 193 (gene ontology) and 43 (gene expression). All of these L1 regularization methods could get subsets of features from all possible drug features. L1SVM selected the smallest number of features from all feature profiles, while SCCA extracted the largest number of features in total. Multi-LRSL selected more features than L1LOG and L1SVM but less features than SCCA except for the chemical substructures. From the venn diagrams in Fig. 3, it is observed that the most features selected by L1LOG and L1SVM overlap with the features selected by multi-LRSL and SCCA. There are many features shared by SCCA and multi-LRSL as well as lots of features specifically selected by these two methods. Furthermore, the numbers of features selected by L1LOG and L1SVM from different feature profiles are quite uneven compared to the other two methods. There are much fewer features selected by L1LOG and L1SVM from the gene expression profile than the other feature types (as shown in Fig. 3 and Additional file 1: Figure S5).

To further illustrate the feature selection property of our algorithm, we calculated the correlations between the drug features from both the same and different feature profiles using the original feature matrices, then we computed the correlations between the feature coefficients obtained by multi-LRSL. It is observed that the correlations between the drug feature coefficients tend to increase as the correlations between the original features become larger (Additional file 1: Figures S6 and S7). It is implied that multi-LRSL could select groups of highly correlated features both within and between multiple feature profiles. This property is similar to elastic net [50]. It should be an advantage for the problem of side effect prediction as there are many important but highly correlated features. Besides, it is realized that strongly correlated side effect labels could be associated with similar drug features. In Additional file 1: Figure S8, the affinity matrices of side effect labels calculated from the feature coefficients of multi-LRSL are visualized. All of these matrices are quite consistent with the affinity matrix of side effect labels calculated from the drug-side effect relation matrix. Thus, the proposed method could capture both the associations between drug features and the correlations between side effect labels.

**Fig. 4:**
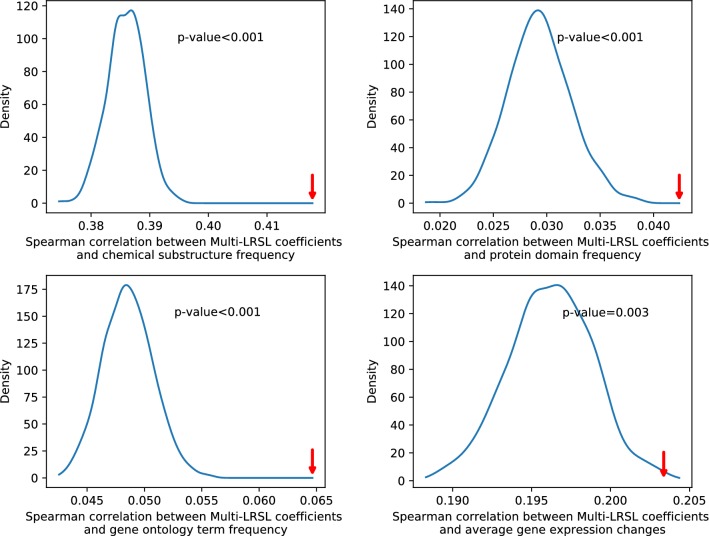
The average Spearman’s correlation between the feature coefficients learned by multi-LRSL and feature data extracted from CTD for the same side effect is significantly bigger than random samples. The blue lines represent the density estimates for the averages of correlation coefficients of 1000 random samples. For each random sample, the average correlation is calculated with the same number of pairs of randomly selected feature coefficients and CTD feature data. The red arrows indicate the positions of the average correlation coefficients between paired feature coefficients and feature data (the frequency of features for chemical substructures, protein domains and gene ontology terms and the averages of gene expression changes). The p-values are estimated by Monte-Carlo test

Next, we tested the stability of our algorithm for feature selection by training the model with random division of drugs. It is shows that the feature coefficients are stable when different subsets of drugs are used for training (Additional file 1: Figure S9). Furthermore, we intend to examine whether the selected features are relevant to the side effects. However, there are very few systematic records for the relationships between drug features and side effects. Thus, we try to verify these predicted associations by examining whether they are compatible with the information from independent data source. For this purpose, the disease terms in CTD [51] which overlapped with the side effect terms in this work were gathered. In CTD, the disease terms are associated with various chemicals and genes. The chemicals and genes labelled with ‘marker/mechanism’ correlate with the disease or participate in the etiology of the disease. We collected these chemicals and genes related to the disease terms which overlapped with side effect terms. Then, the substructures and the gene expression changes of these chemicals, the protein domains and the gene ontology terms of these genes were extracted. The occurrence frequency of chemical substructures, protein domains and gene ontology terms were calculated for each disease term according to its related chemicals and genes to form the corresponding features from CTD. The gene expression changes were averaged across the chemicals related to a disease term to form the gene expression features from CTD. The data from CTD could be considered as the additional evidence to support the relationships between features and side effects. In our model, we assume that a feature with a coefficient that is large in magnitude is predictive for the presence of a side effect. We assume that the features from CTD will also have large magnitude and match the sign of the coefficients. Thus, the correlations between the coefficients and the CTD features could be utilized to assess the consistency between the predicted feature-side effect association and the information in CTD. We calculated the Spearman correlations between the coefficients learned by multi-LRSL and the features obtained from CTD for each side effect. It is found that the average correlation is significantly bigger than the correlations of randomly paired coefficients and features (Fig. 4, $\hbox {p-value}<0.05$). Together with the previous results, it is suggested that the proposed method could help select drug features related to side effects.

### Predicting new drug-side effect associations

**Fig. 5:**
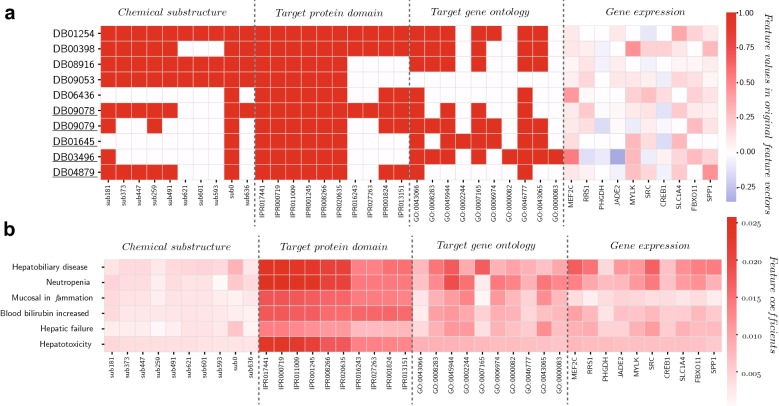
The prediction results for hepatotoxicity. **a** The X axis represents the features with the largest coefficients for hepatotoxicity (10 features from each feature profile). The Y axis represents the top-ranked predicted drugs (5 test drugs without any known side effects, and 5 drugs with known side effects but without record for hepatotoxicity. The DrugBank IDs of the drugs with known side effects are underlined). Here, the colours on the heat-map represent the values of the selected features in the feature vectors of these drugs. **b** The X axis represents the features with the largest coefficients for hepatotoxicity (10 features from each feature profile), and the Y axis represents the side effects most frequently co-occurred with hepatotoxicity. The colours on the heat-map represent the values of the coefficients learned by multi-LRSL for each side effect. In both (**a**) and (**b**) different types of features are separated by grey dash lines

To illustrate the utility of the proposed method for predicting side effects, we collected 320 drugs from DrugBank which were not included in the 501 drugs used for model construction. The DrugBank identities and names of these drugs are available at (Additional file 2: Table S7). We choose these drugs because all types of features could be obtained for them but they don’t have any side effect records in SIDER. In order to predict the side effect labels of these drugs, the features of them were retrieved as previously described. Then, the feature coefficient matrices $G_{p}\hbox {s}$ were learned by our model with all the 501 drugs as the training data. We predicted side effects of these new drugs by $\sum _{p=1}^m{\theta _pX^{new}_pG_p}$. $X^{new}_p$ is the *p*th feature matrix of these new drugs. Moreover, we inferred the missing labels of the training drugs with the predicted label matrix *F* (see methods section for more details). To give insights into the prediction results of our method, some examples are provided here. Hepatotoxicity is an important clinical adverse event that could cause hospitalizations and withdrawal of drugs. In Fig. 5a, 10 predicted drugs for hepatotoxicity (5 top-ranked new drugs, 5 top-ranked drugs in the training data without record of hepatotoxicity) are picked. The top-ranked features for hepatotoxicity (10 features with the highest coefficients of each feature type) are selected. The values of the top-ranked features in the original feature vectors of the 10 drugs are shown as heat-map. In the predicted drugs, dasatinib (DB01254), a selective tyrosine kinase receptor inhibitor for treatment of chronic myelogenous leukemia, was reported to induce live dysfunction [52]. Nintedanib (DB09079) also showed hepatotoxicity in a clinical trail [53]. It is observed that among the 10 top-ranked chemical substructures, 9 substructures (except sub0: $\ge 4\,\hbox {H}$) are enriched in the drugs with hepatotoxicity (Fisher’s exact test, p-value 1e−4). Among the selected protein domain features, there are 8 domains related to protein kinase, and 6 of them are present in the targets of all these drugs. This is in accordance with the previous study that many tyrosine kinase inhibitors have been found to be hepatotoxic [54]. The selected gene ontology features are mainly involved in apoptosis and cell proliferation. These biological processes were also related to hepatotoxicity by previous studies [55, 56]. It is observed that all drugs show a similar pattern of disturbance to the expression level of MEF2C. MEF2C was reported to regulate the activation of hepatic stellate cells and play a key role in hepatic fibrosis, a pathological response to live injury [57]. In Fig. 5b, the side effects that most frequently co-occur with hepatotoxicity show similar patterns of top-ranked feature coefficients. Some of these side effect terms are semantically related to hepatotoxicity, such as hepatobiliary disease and hepatic failure, while the other side effects may be similar to hepatotoxicity in production mechanisms. For example, drug-related neutropenia could be caused by cytotoxic effect on cell replication [58]. Mucosal inflammation could be regulated by tyrosine kinases related signal pathways [59]. In Additional file 2: Table S8, the prediction results of another two side effects, renal impairment and acute myocardial infarction are provided as additional examples. Alvimopan (DB06274) is ranked 3th for acute myocardial infarction. This toxic effect has been proved by clinical observation [60]. The selected features may also give some hints about these side effects. DAXX is ranked 1st for acute myocardial infarction in the gene expression features, and a study showed that DAXX may participate in myocardial ischemia/reperfusion-induced cell death [61]. The relationship between SRC (ranked 2th) and renal damage was also implied by previous study [62]. The above instances suggest that the proposed method could predict novel drug-side effect associations and select important drug features.

## Discussion

Side effects are unintended impacts of drugs on human bodies. It is important to develop efficient methods for identifying potential side effects. In this work, we propose a novel multi-view and multi-label learning algorithm to predict the side effects of small molecular drugs and select relevant features. The advantage of the proposed method is demonstrated by systematic comparison with other computational methods and some examples of application.

The proposed method could integrate multiple types of features for side effect prediction. The rationality behind integration of multi-view data is that side effects are the results of complex interactions between drugs and biological systems. Drugs could interact with both intended therapeutic targets and unintended off-targets. While both types of targets could be associated with side effects, the full list of drug targets is still not available. Furthermore, targets could impact on the activities of various biological processes and pathways after binding with drugs. On the other hand, chemical structures determine how drugs interact with targets. Gene expression changes induced by drug reflect the overall biological effects of drug-target interactions. Thus, combination of chemical structures, gene expression and target information could provide a relatively complete description of drug bioactivity for side effect prediction. In this study, we show that there is consistent and complementary information related to side effects in these heterogeneous data. Our results also show that integration of multi-view data improves the performance of prediction. Therefore, data fusion for side effect prediction is reasonable and necessary.

In this study, the graph Laplacian regularization of the predicted label matrix encourages the preservation of the local geometric structures of the feature space and lets the drugs with similar features have similar side effect labels. The graphs constructed from four types of features are combined to explore the complementary information from different sources. This strategy for heterogeneous data integration is similar to the previous works [3, 32]. However, there are also some special improvements for the side effect prediction problem. In the work of Shi et al. [32], different types of features were concatenated into a long vectors in the least square loss term. This will increase the computational complexity for updating coefficient matrix as the total dimension of features is high in the side effect prediction problem (as noted by the algorithm complexity analysis in the additional material). They also selected the features associated with the larger rows in the coefficient matrix by $l_{2,1/2}$-matrix norm [32]. This will keep a group of features from all feature profiles to predict all labels. In this work, the feature matrices and the coefficient matrices are separate in both the least square loss terms and the graph Laplacian regularization terms. The L1 penalties induce the element-wise sparsity of the coefficient matrices [63]. Thus, our method could select different features from each feature profile simultaneously for different labels and reduces the computational cost. Moreover, in this work, the information of the known labels is used differently from our previous work for drug-disease association prediction [3]. Instead of transforming the label similarity to drug similarity, the correlations between side effects labels are explicitly encoded by an additional graph Laplacian matrix that regularizes the feature coefficient matrices. The regularization makes the strongly correlated labels share more relevant features. Altogether, the proposed model could not only fuse multi-view data but also select label specific features with the consideration for label correlations.

The dimensions of chemical and biological features of drugs are usually high. Feature selection is beneficial for side effect prediction, because it could reduce computational cost and prevent overfitting by excluding irrelevant features [64]. L1SVM, L1LOG, SCCA and our model introduce sparsity by L1-regularization. There is a trade-off between the feature sparsity and the prediction performance (Additional file 1: Figure S10). Although L1SVM and L1LOG selected less features, the correlations between features from multiple feature profiles could be missed by these two methods [50]. The correlations between side effect labels were also not taken into consideration. For SCCA, features and side effects appeared many times in multiple canonical components, which made the relationships between features and side effects less obvious. The proposed method fused heterogeneous data and considered the correlations between side effect labels. Thus, it could select features from multi-view data and associate every side effect with special drug features. As a result, our method could be utilized just for feature selection, and a separate classifier could be constructed with the selected features as the input to further improve the prediction performance. In that situation, the selection stability analysis should be conducted. Furthermore, the linear regression with L1 regularization in the model could increase the transparency of the model, that is, users could recognize which features are important for the model to make the predictions. However, it should be noticed that the model could not reveal the causal relationships between drug features and side effects. Users should be cautious about the meaning of the selected features. Feature selection sometimes could help researchers generate new hypothesises about the relationships between the selected features and the class labels. For this purpose, it is important to use interpretable features as input variables. The protein domain, gene ontology and gene expression features in this work could be more interpretable than chemical fingerprints. It is desirable to explore new forms of input in order to make the selected chemical features more meaningful in future work.

Although the proposed method shows the advantage of information fusion for side effect prediction, there are also some limitations in current work. First, the number of samples used for training is crucial for prediction and feature selection. But collecting multiple types of data is difficult and some features may not be available for some drugs. For example, not all the drugs in SIDER have the records of protein targets or gene expression data. This leads to a smaller data set for training the algorithm. However, our method is scalable, it could take either single or multiple feature profiles as input. Additionally, like the previous study [32], our model is based on graph Laplacian regularization. The model could be extended to a semi-supervised method [29]. Semi-supervised learning could utilize unlabelled data to promote the prediction performance. It may alleviate the problem of the limited number of labelled samples in side effect prediction. Second, there are some discrepancies between multiple data types. For example, some targets of drugs may be missed and gene expression data may contain lots of noise. The discrepancy could impair the performance of prediction. Thus, it needs methods that could handle disparity and noise in the future work. Thirdly, the side effect labels of drugs may be also missing and noisy. For example, FAERS contains a lot of drug-side effect associations which are absent in SIDER. There may be also some false positive labels in SIDER [20]. Some side effects are non-specific and don’t have causal relationships with drug features [65]. The missing and noisy labels could bring negative impact on side effect prediction. Because which side effect labels are missing is not known, all unobserved values are set to 0 in the label matrix, this could aggravate the bias of the model. The missing labels also aggravate the class imbalance and make the estimation of label correlations inaccurate. In the proposed model, we used the predicted label matrix *F* to approximate the true label matrix, and refined the label correlation matrix during optimization. This could alleviate the influence of missing label, but more sophisticated algorithms are needed to solve this problem in future study. For example, positive-unlabelled learning may alleviate the influence of the noisy negative labels [66].

## Conclusions

In this work, we develop a novel computational method for predicting drug side effects. The proposed method could fuse multi-view data and explore the correlations between side effects. It could not only improve the performance of prediction but also select multiple types of features related to side effects. As a result, our method could be potentially used as an effective computational tool for recognizing patterns of side effect related features from various sources of data. In this way, the method could provide instructive information for drug development by mining heterogeneous data.

## Supplementary information

**Additional file 1.** Analysis of the proposed method and additional figures.

**Additional file 2.** Additional tables.

## Data Availability

All data are available together with the code for the proposed method at https://github.com/LiangXujun/multi-LRSL.
