# Supplementary material for: Learning important features from multi-view data to predict drug side effects
Source: J Cheminform. 2019 Dec 16;11:79. doi: 10.1186/s13321-019-0402-3 (PMC6916463; doi:10.1186/s13321-019-0402-3)
Supplement: Supplementary file 1 — Additional file 1. Analysis of the proposed method and additional figures. [file 13321_2019_402_MOESM1_ESM.pdf]

Supplementary Material

1 Implementation details of the comparison methods

L1LOG and L1SVM were implemented with the python package 'Liblinear'[1]. The cost parameters C of L1LOG and L1SVM were searched using cross validation and set to 50 for L1LOG, 10 for L1SVM. As many side effect labels were imbalanced, the class weights were set as the reciprocal of the number of positive or negative samples in each class. PCR was implemented with scikit-learn, after applying PCA to the concatenated feature matrix, the first 400 principal components(determined by cross-validation) were used as the input of logicist regression. For SCCA, 80 canonical components were kept, the penalty parameter '*penaltyx*' and '*penaltyy*' were set to 0.3(all parameters were determined by cross-validation). For kernel regression, gaussian radical basis function(RBF) was applied to each feature profile. The same weight was used for each feature kernel in kernel integration. The parameter *alpha* of KernelRidge function in scikit-learn was learned and set to 1.

2 Analysis of multi-LRSL algorithm

2.1 Algorithm complexity

Here we analyse the algorithm complexity of multi-LRSL. Suppose the number of training drugs is n, the dimension of the p-th feature profile is d_p, the number of side effects is l. The complexity for Laplacian matrix L_p construction is $\mathcal{O}(n^2 d_p)$. In each iteration, the optimization of F has the complexity of $\mathcal{O}(n^3 + nld_p)$, the complexity for learning R is about $\mathcal{O}(l^2 d_p)$. For training data used in this study, d_p and l are much larger than n, the complexity for learning G_p is about $\mathcal{O}(ld_p^2)$. As a result, the most complex part in each iteration is about $\mathcal{O}(l^2 d_p + ld_p^2)$.

2.2 Parameter Sensitivity analysis

There are several parameters in multi-LRSL. The parameter k is empirically set to $0.01n$ for the knn graph of drugs and $0.01l$ for the knn graph of side effect labels. γ is empirically set to 2 as the previous work[2, 4, 3]. The rest parameter μ, λ, α, β are learned using grid search and cross-validation on the training data. As show in Figure S10, the sample-AUC score is sensitive to the values of μ and β, but doesn't change too much as the values of λ and α vary. It is observed that the performance of the model is decreased as the parameter β becomes larger than 0.1. Because larger β means less features are selected, it is suggested that there is a trade-off between the number of selected features and the prediction performance. Finally, the values of μ, λ, α and β are set to 0.1, 1, 1 and 0.01 respectively.

References

[1] Rong-En Fan, Kai-Wei Chang, Cho-Jui Hsieh, Xiang-Rui Wang, and Chih-Jen Lin. Liblinear: A library for large linear classification. *J. Mach. Learn. Res.*, 9:1871–1874, June 2008.

[2] Xujun Liang, Pengfei Zhang, Lu Yan, Ying Fu, Fang Peng, Lingzhi Qu, Meiying Shao, Yongheng Chen, and Zhuchu Chen. Lrssl: predict and interpret drug-disease associations based on data integration using sparse subspace learning. *Bioinformatics (Oxford, England)*, 33:1187–1196, April 2017.

[3] Caijuan Shi, Qiuqi Ruan, Gaoyun An, and Chao Ge. Semi-supervised sparse feature selection based on multi-view laplacian regularization. *Image and Vision Computing*, 41:1 – 10, 2015.

[4] J. Yu, M. Wang, and D. Tao. Semisupervised multiview distance metric learning for cartoon synthesis. *IEEE Transactions on Image Processing*, 21(11):4636–4648, November 2012.

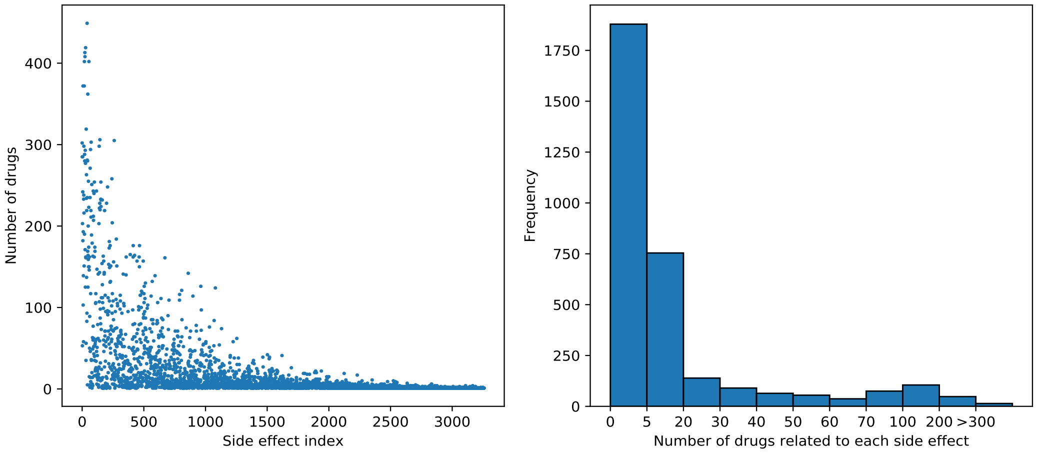

Figure S1: Distribution of drug-side effect associations. The left panel shows the number of drugs related to each side effect, and the right panel is the histogram of the frequency of side effects associated with different numbers of drugs.

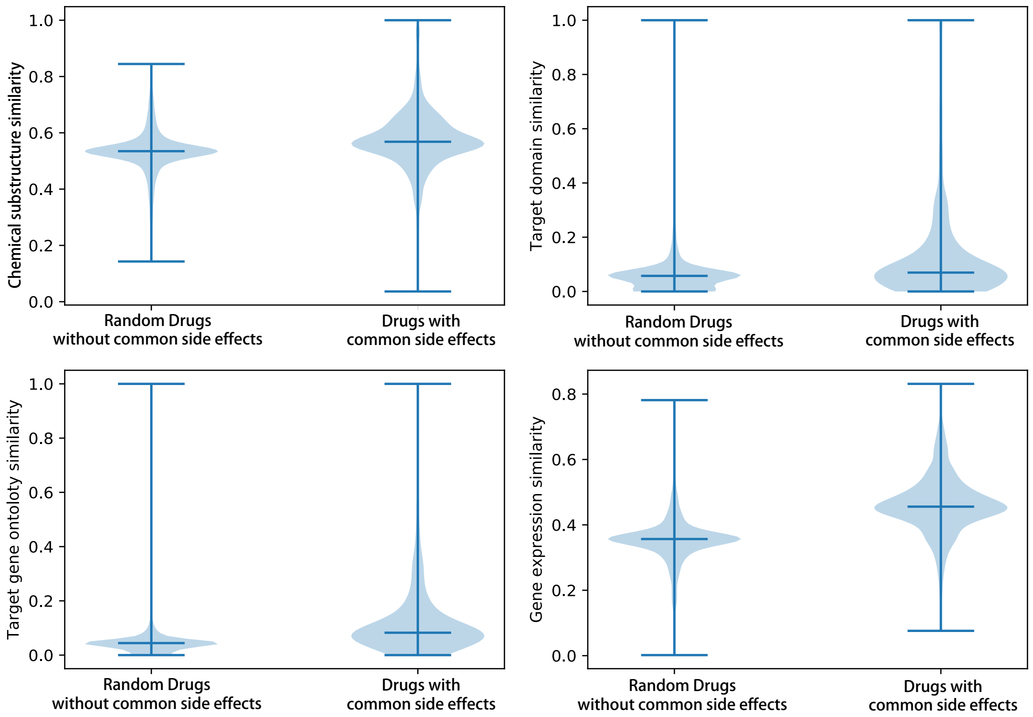

Figure S2: The average similarity of drugs with common side effects is larger than the average similarity of the same number of randomly selected drugs without common side effects. The four panels correspond to the violin plots of the drug similarities measured by four different feature types. All comparisons are statistically significant(rank sum test, p-value$<$0.001)

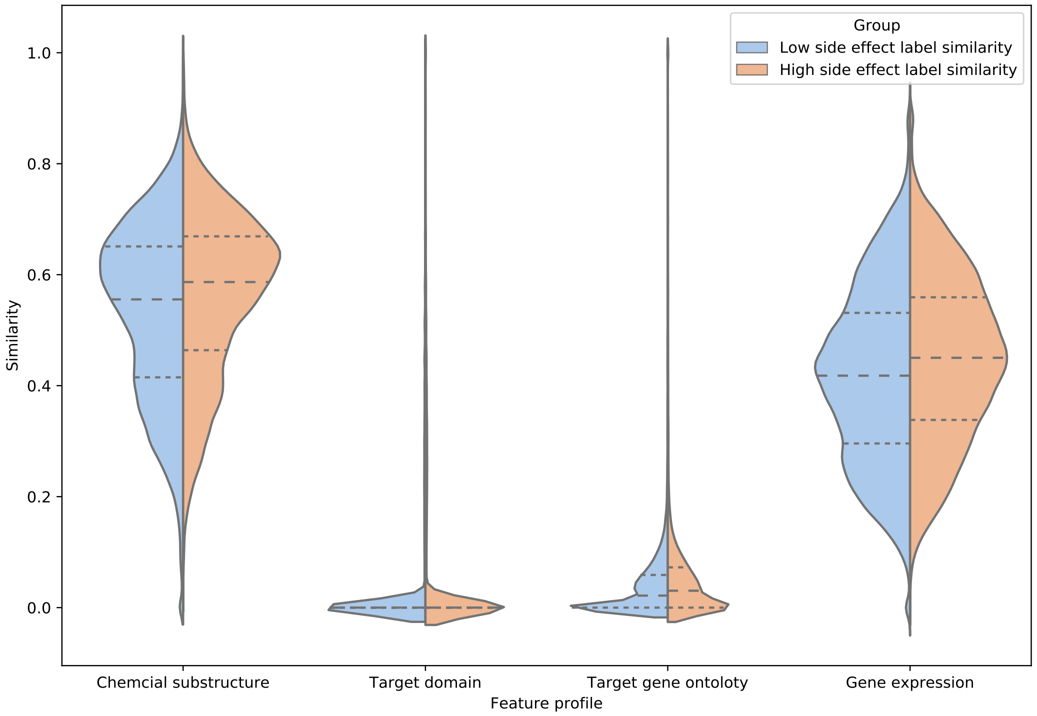

Figure S3: Drugs with stronger side effect similarity are also more similar in different feature profiles. All comparisons are statistically significant(rank sum test, p-value<0.001). The dashed lines denote the positions of quartiles.

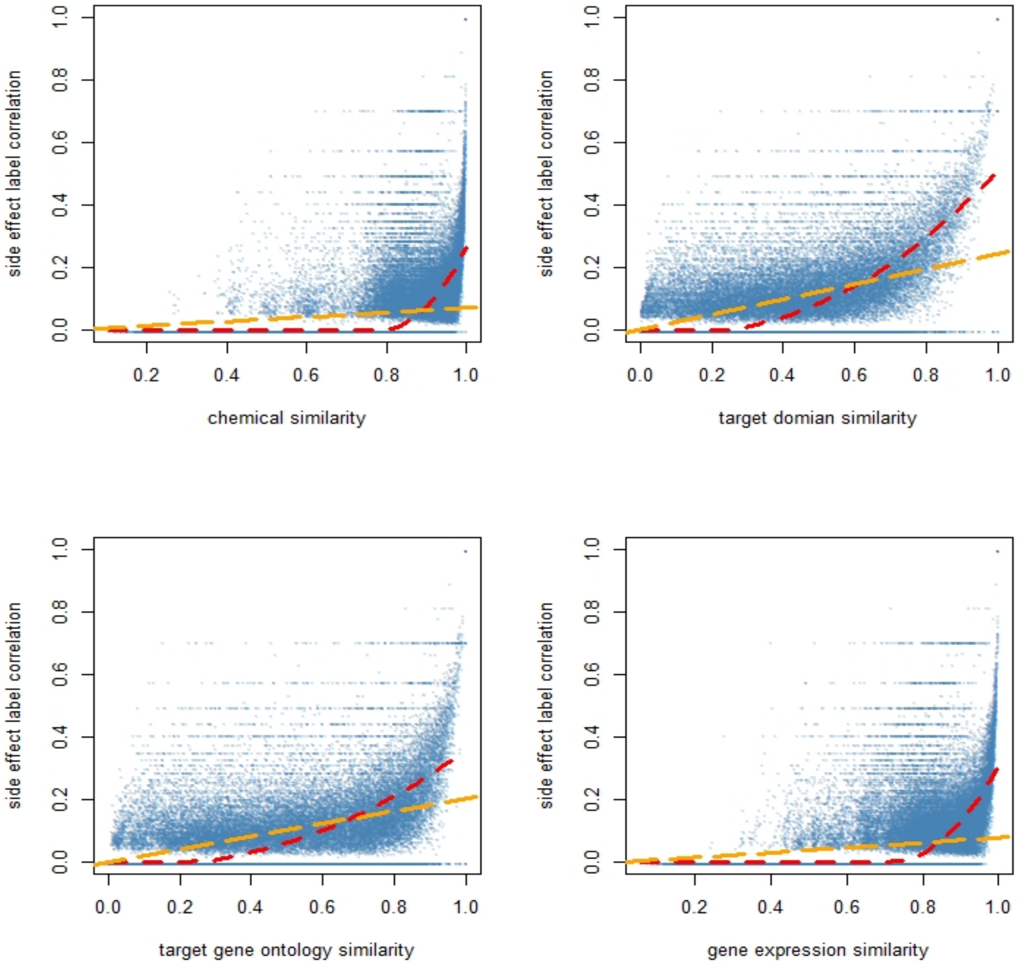

Figure S4: The correlations of side effect labels positively correlate with all types of feature similarities. The orange lines represent the ordinary least squares models, while the red lines are the local polynomial regressions of the points.

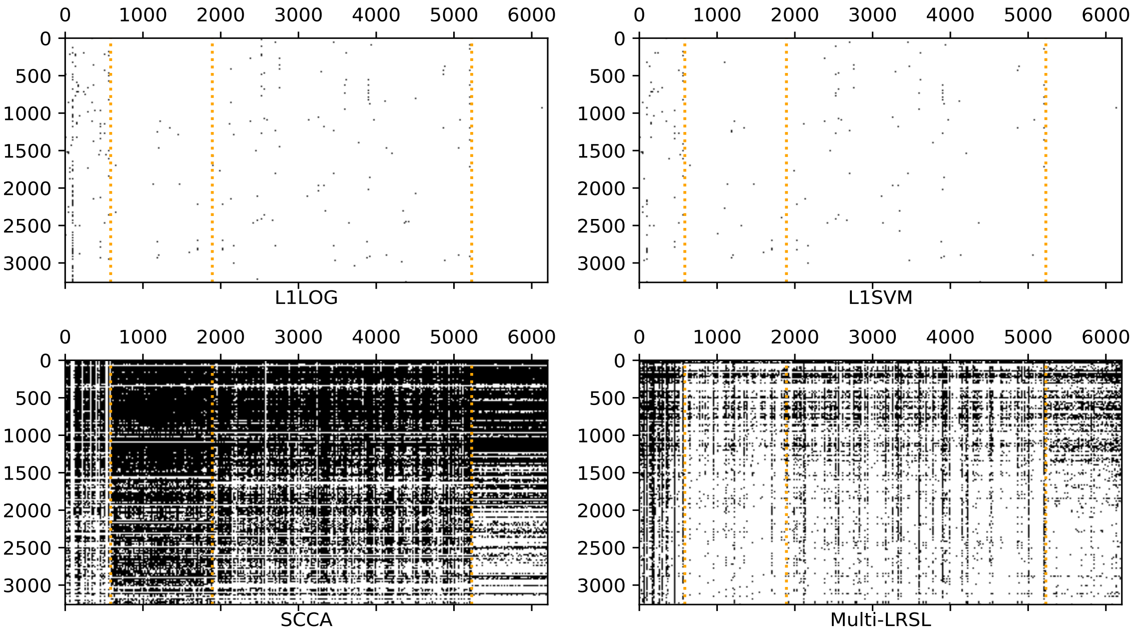

Figure S5: Visualizing the feature coefficient matrices of different algorithms. The horizontal axes indicate different drug features, the vertical axes indicate different side effect labels. The orange vertical lines separate the matrices into the blocks of different drug feature profiles.(From left to right in each panel: chemical substructure, target domain, target gene ontology, gene expression)

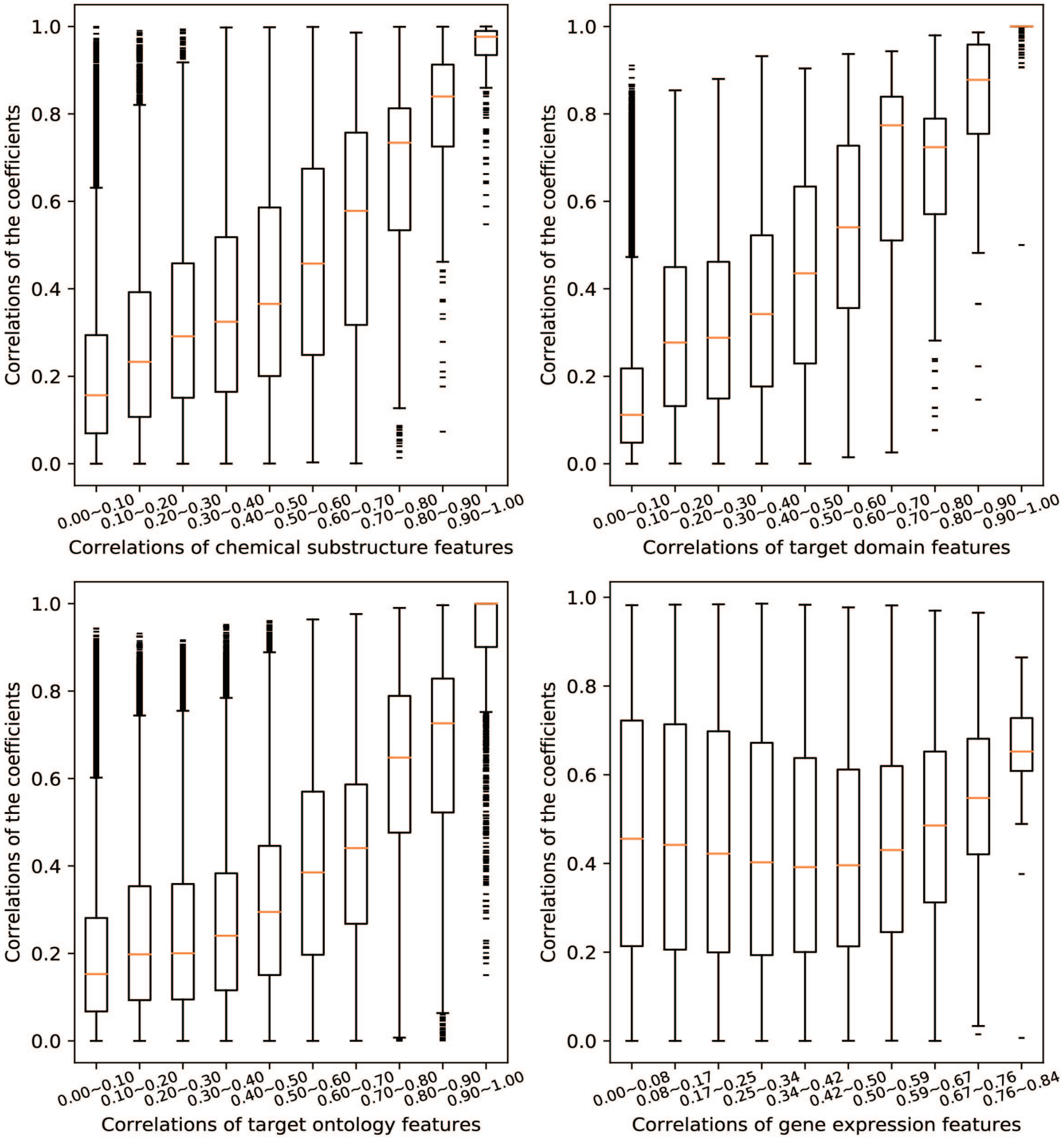

Figure S6: Feature coefficient correlations get bigger as the drug feature correlations increase in the same feature profile. The absolute values of the pearson correlation coefficients are calculated by using the relationships between features and drugs, then the values are divided into ten equal-width bins. For feature pairs in each bin, the box plots show the distribution of the absolute values of the correlations between the feature coefficients.

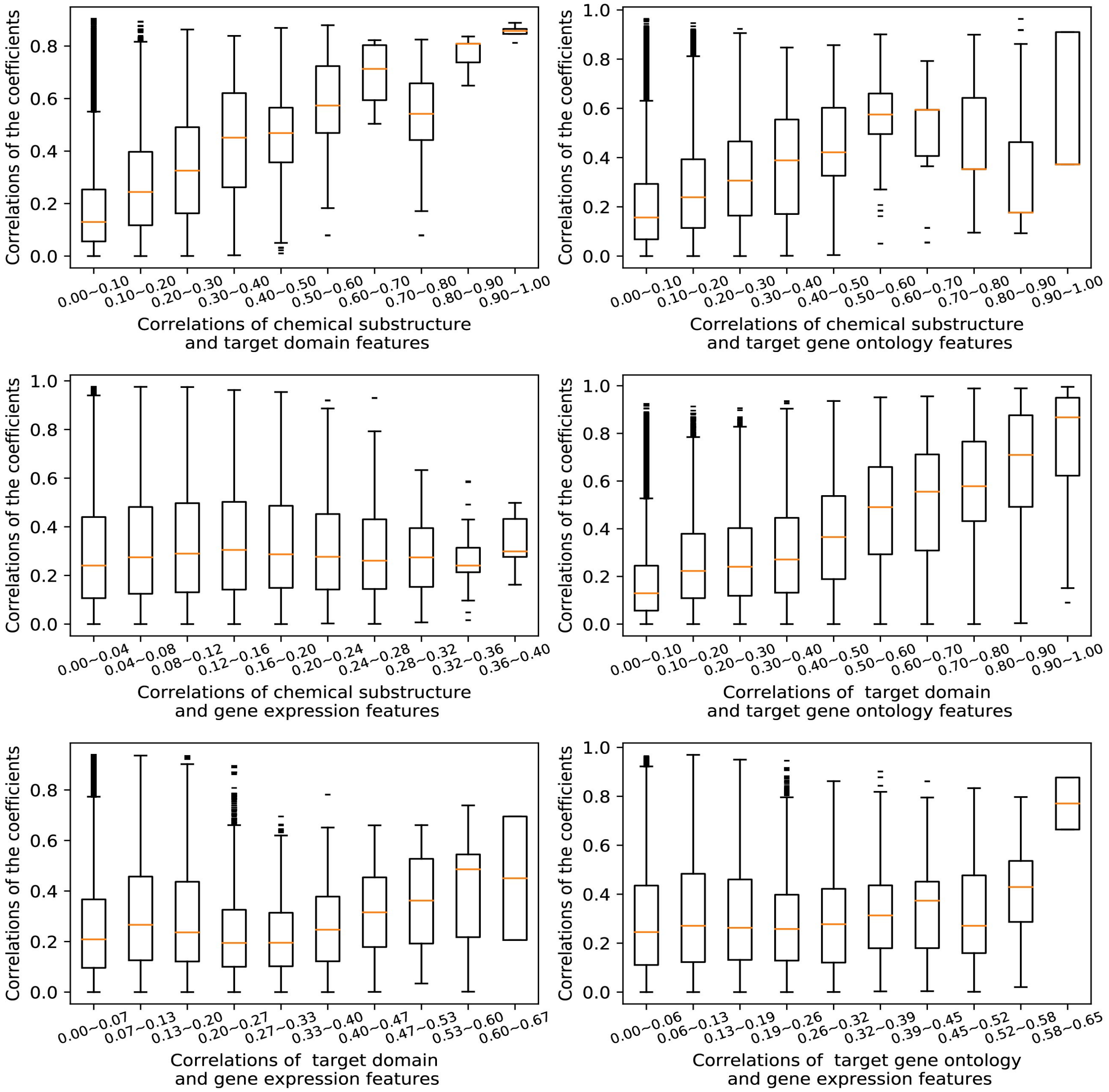

Figure S7: Feature coefficient correlations get bigger as the drug feature correlations increase between different features profiles. The absolute values of the pearson correlation coefficients are calculated by using the relationships between features and drugs, then the values are divided into ten equal-width bins. For feature pairs in each bin, the box plots show the distribution of the absolute values of the correlations between the coefficients of features from two different feature profiles.

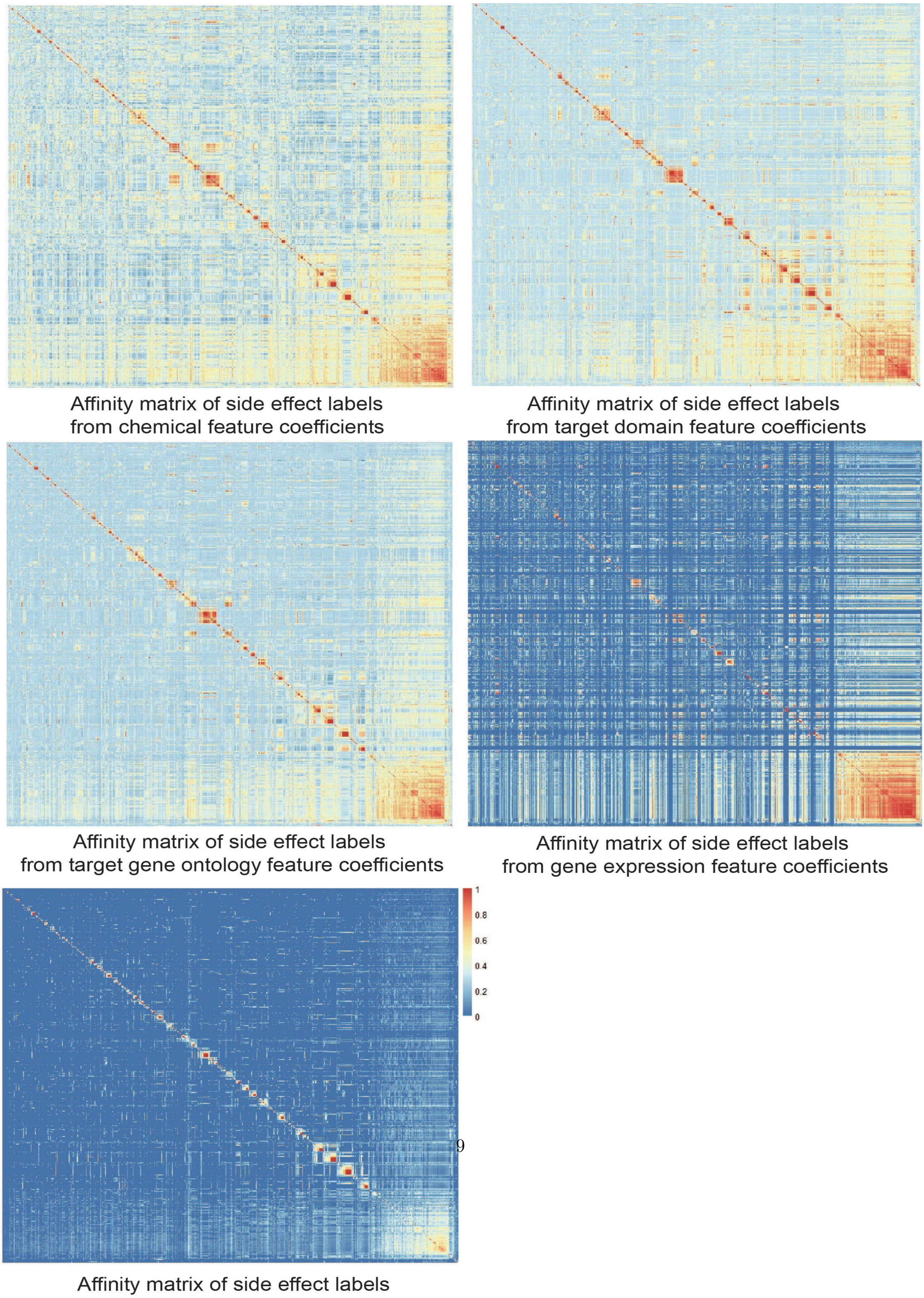

Figure S8: The affinity matrix of side effect labels is similar with the affinity matrices calculated from different feature coefficients.

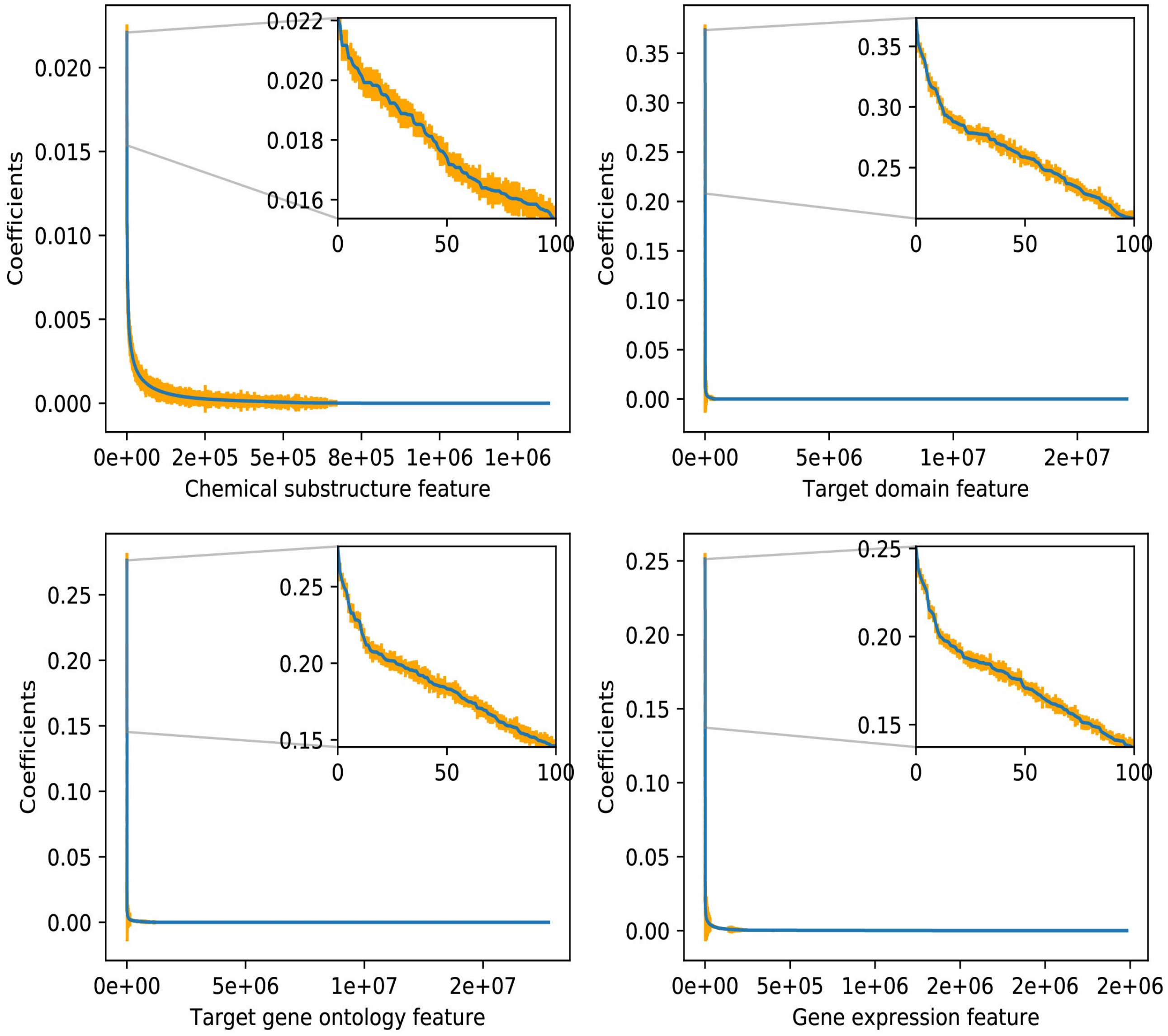

Figure S9: Stability of feature selection using random drug subsets. The drugs were randomly divided to ten subsets, at each time, one subset was excluded and the left drugs were taken as the training set to calculate the coefficients of features. The averages and standard deviations of feature coefficients are shown as line charts with error bars, the inserted plots show the 100 biggest coefficients.

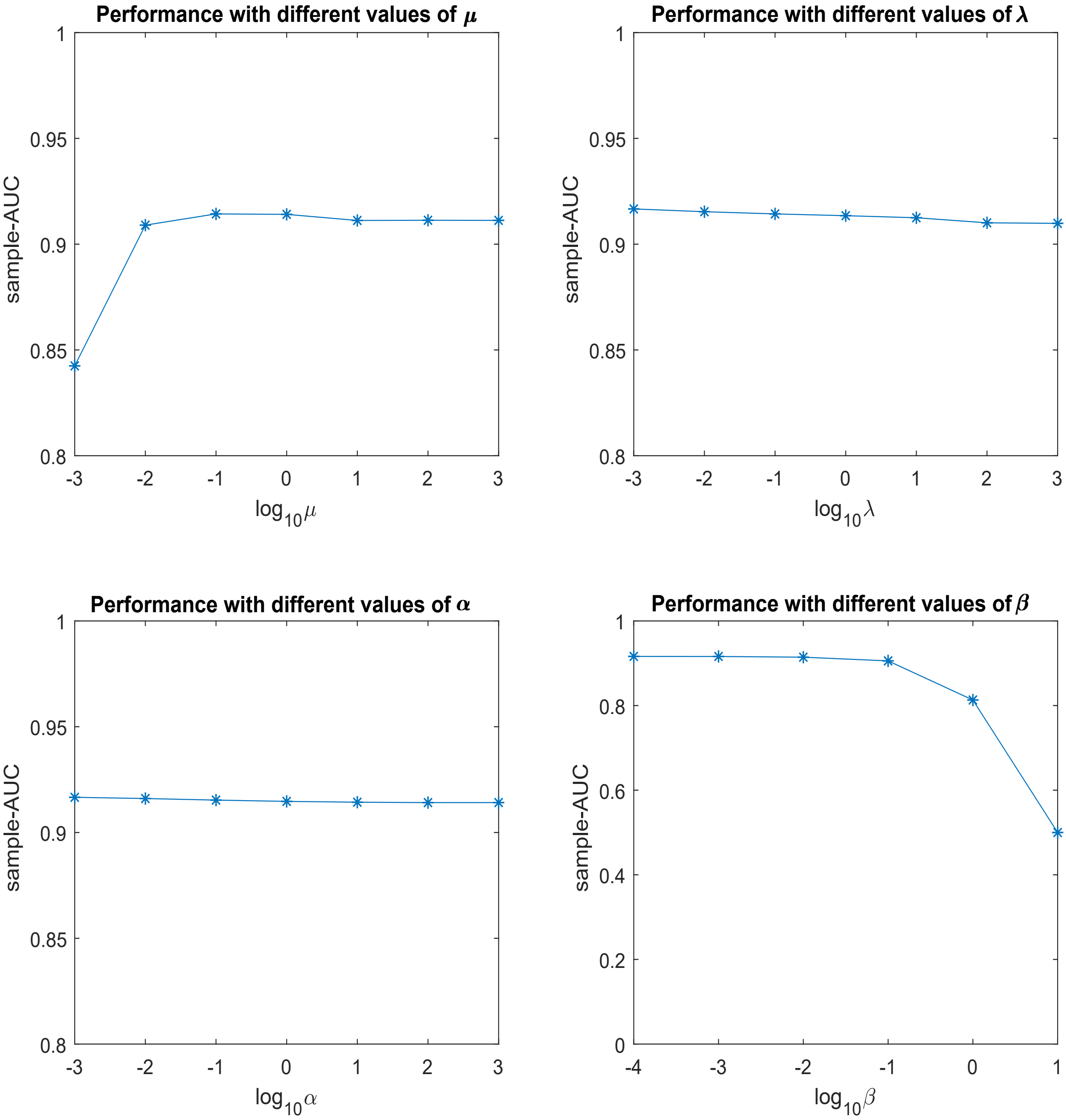

Figure S10: Effect of model parameters on the performance of multi-LRSL in side effect perdition task.
